# Predicting Interactions between Common Dolphins and the Pole-and-Line Tuna Fishery in the Azores

**DOI:** 10.1371/journal.pone.0164107

**Published:** 2016-11-16

**Authors:** Maria João Cruz, Gui Menezes, Miguel Machete, Mónica A. Silva

**Affiliations:** 1 Departamento de Oceanografia e Pescas, Universidade dos Açores, 9901–862, Horta, Portugal; 2 MARE–Marine and Environmental Sciences Centre and Centre of the Institute of Marine Research (IMAR)–University of the Azores, 9901–862, Horta, Portugal; 3 Biology Department, Woods Hole Oceanographic Institution, Woods Hole, Massachusetts, United States of America; University of Minnesota, UNITED STATES

## Abstract

Common dolphins (*Delphinus delphis*) are responsible for the large majority of interactions with the pole-and-line tuna fishery in the Azores but the underlying drivers remain poorly understood. In this study we investigate the influence of various environmental and fisheries-related factors in promoting the interaction of common dolphins with this fishery and estimate the resultant catch losses. We analysed 15 years of fishery and cetacean interaction data (1998–2012) collected by observers placed aboard tuna fishing vessels. Dolphins interacted in less than 3% of the fishing events observed during the study period. The probability of dolphin interaction varied significantly between years with no evident trend over time. Generalized additive modeling results suggest that fishing duration, sea surface temperature and prey abundance in the region were the most important factors explaining common dolphin interaction. Dolphin interaction had no impact on the catches of albacore, skipjack and yellowfin tuna but resulted in significantly lower catches of bigeye tuna, with a predicted median annual loss of 13.5% in the number of fish captured. However, impact on bigeye catches varied considerably both by year and fishing area. Our work shows that rates of common dolphin interaction with the pole-and-line tuna fishery in the Azores are low and showed no signs of increase over the study period. Although overall economic impact was low, the interaction may lead to significant losses in some years. These findings emphasize the need for continued monitoring and for further research into the consequences and economic viability of potential mitigation measures.

## Introduction

Direct interactions between marine mammals and fishery operations pose a threat to the sustainability of many marine mammal populations and could be detrimental to fisheries. These interactions may result in the injury or death of marine mammals, damage to the fishing gear, reduced catches and increased time spent in fishing operations [1–3].

World tuna catches have been increasing constantly and rapidly [4–6]. Industrial fishing for tunas began in the 1950’s and global catches increased from 0.2 million t in 1950 to over 4 million t in 2013 [4, 7]. The total catch of tuna and tuna-like species accounted for nearly 9% of global marine fisheries catches by landed weight [8]. The principal tuna market species were primarily taken from the Pacific (70.5% in 2008), followed by the Indian Ocean (19.5% in 2010), the Atlantic and the Mediterranean Sea (10% in 2010) [9]. Purse seine, pelagic longline and pole-and-line fisheries are the primary commercial fishing methods for catching tunas [4, 5].

In the eastern tropical Pacific Ocean (ETP) the purse seine tuna fishery take advantage of the association of large yellowfin tuna (*Thunnus albacares*) with small dolphins, particularly spotted dolphins (*Stenella attenuatta*), spinner dolphins (*Stenella longirostris*) and common dolphins, to locate tuna schools which are then encircled together with large herds of dolphins. Consequently, several hundreds of thousands of dolphins were incidentally killed per year between the 1960s and 1990s [10–12]. The provisions to reduce dolphin mortality included in the U.S. Marine Mammal Protection Act passed in 1972, led to a 99% decline in dolphin bycatch [13]. Despite this reduction this fishery still kills about 1000 dolphins every year [14]. In the western and central Indian Ocean, the most important interaction between cetaceans and the tuna fishery may be in tuna gillnets with about 60,000 small dolphins taken annually [15]. In the northeast Atlantic, albacore tuna (*Thunnus alalunga*), striped dolphins (*Stenella coeruleoalba*) and common dolphins were often caught together in the same nets during commercial seining operations [16, 17]. Unlike the former fishing gears, pole-and-line fishing is highly selective with low bycatch rates of cetaceans or other non-target species [6, 15].

The pole-and-line tuna fishery is one of the most important fisheries in the archipelago of the Azores representing approximately 60% of total landings and 39% of the economic revenue from the fishing activity within the last five years [18]. Tunas are caught with pole-and-line, usually using water spray and live bait. The predominant species in the catch are bigeye tuna (*Thunus obesus*), present from April to June, and skipjack (*Katsuwonus pelamis*), usually targeted from June to October. Albacore, yellowfin and bluefin tuna (*Thunus thynnus*) are captured in smaller quantities [19, 20]. Live bait consists of small pelagic fish mainly European pilchard (*Sardina pilchardus*) and blue jack mackerel (*Trachurus picturatus*) [20, 21].

In the Azores, dolphins were previously captured both for human consumption and baitfish [20]. The tuna fishery has been monitored by the Azorean Fisheries Observer Programme (POPA) (www.Popaobserver.org) since 1998 to ensure the “dolphin safe” label to tuna caught in the Azores. The label implies that the fishery is monitored and there is no chasing, capture or handling of dolphins during an entire tuna fishing trip. POPA monitors about 50% of the tuna fleet and, in general, over 50% of the tuna catches (minimum required to certify the fishery as “dolphin safe”) [19, 21]. Trained observers working on board tuna fishing boats collect information about the fishery, the catch and interactions with non-target species.

Silva *et al*. [22, 23] studied the interaction between cetaceans and the tuna fishery in the Azores. This fishery shows low rates of capture of cetaceans and no incidental mortality, with all animals released alive and apparently unharmed by cutting the fishing line. However, cetaceans were reported to interact with the fishing by sinking the tuna schools and/or competing with tunas for live bait, resulting in an increase in the proportion of fishing events with no catches and an increase in the duration of fishing operations. Common dolphins were responsible for 77% of the cases of interaction (n = 319), though the authors reported a decreasing trend in interference over the years. Although these studies provided important information to characterize the interaction between small dolphins and the pole-and-line fishery in the Azores, we still do not understand the factors contributing to the interaction. Also, reliable estimates of catch loss associated with dolphin interaction are urgently needed to assess impacts from the interaction. Understanding which conditions favour cetacean-fisheries interactions and the consequences for cetacean populations and for the fisheries is crucial for informing management decisions.

To comprehend spatial and temporal patterns and effects of cetacean interactions with the pole-and-line fishery in the Azores we analysed data collected by POPA within the Exclusive Economic Zone (EEZ) of the archipelago between 1998 and 2012. We focused only on interactions with the common dolphin as this was the species involved in most interaction reports. The specific objectives of this study were to (i) verify the decreasing trend in interaction rates reported by Silva *et al*. [23] using a longer time series (ii) understand which environmental and fisheries related factors influence the frequency of interactions (iii) and quantify the effect of this interaction on the catches of each tuna species.

## Methods

### Study Area

The Archipelago of the Azores (Portugal) is located in the middle of the North Atlantic, between 37° and 41° N and 25° and 31° W, crossing the Mid-Atlantic Ridge [24]. The archipelago consists of nine volcanic islands and is divided into three groups (eastern, central and western) extending over 600 km. The study area comprises a wide range of habitat types, including narrow island shelves, steep island slopes, shallow seamounts, submarine canyons, and vast areas of abyssal plain [24]. The tuna fishery generally concentrates around the islands and offshore seamounts showing considerable variations in the geographic distribution of the fishing effort between the years [19, 22].

### Fishery observer data

Data on the tuna fishery and cetacean-fishery interactions for the period 1998–2012 were obtained from the Azorean Fisheries Observer Program (POPA) data base [21]. POPA only monitors vessels exclusively dedicated to tuna fishing. The fleet consists of about 20 vessels over 20 meters long and with crews of 14 to 18 men. The fishery occurs between May and November and each vessel spends an average of 8 days at sea before landing [19, 22]. The fishing activity starts early morning, with the vessels searching for tuna schools with binoculars and using seabirds or floating objects as sighting cues. When a tuna school is encountered, fishers activate the water spray and the live bait is thrown into the water to attract the tunas. Each daily fishing trip may consist of several fishing events that are defined as when fishers are effectively capturing tuna. The number of fishing events per day varies greatly depending on tuna abundance and size of the schools encountered. Successful fishing events may last up to 16 hours but the average duration is about 25 minutes [22].

A single POPA observer is assigned to each vessel for a 30 day period, after which observers rotate among fishing vessels. Observers are required to monitor all fishing events and systematically record information on fishing effort, gear characteristics, catches of tuna and non-target species, presence and interaction of cetaceans, seabirds and turtles. Observers receive training in tuna species identification and estimation of length-weight measurements to determine fork length and the whole weight of individual tuna captured. For each fishing event, the observers count the number of tuna caught and estimate the total weight caught.

Cetaceans were considered to be present during a fishing event if at least one individual was seen within 50 m of the target tuna school. In these cases, the observers recorded the species, number of individuals, behaviour and whether cetaceans interacted with the fishing activity. Cetaceans were considered to interact with the fishing activity either when they frightened the tuna school causing them to “sink” in the water column, when they fed on the live bait, or both. Fishing effort was calculated as the number of fishing events per trip and per vessel.

### Environmental data

The environmental variables selected for this study were depth, distance to shore, sea surface temperature (SST) and a proxy for prey abundance. These variables were selected because they are known to affect the abundance and distribution of many marine taxa, including dolphins, tunas and their potential prey [25–27]. Chlorophyll a was not used in this study since data were not available for the time period prior to 2002.

Depth (m) and distance to shore (km) of each fishing event were calculated in ArcGIS. Depth was obtained from composite bathymetric grids from GEBCO_08 [28], (MOMARGIS v2, DOP/UAz) and multi-beam surveys (GMRT grids). Original grid resolution varied between 50 m and 0.5'. Distance to shore was calculated as the shortest distance between the coordinates point and the closest point on the coastline. SST at each fishing event was obtained from *in situ* measurements made by the vessel echosounder. When SST values were not available for a fishing event, average SST for that month were used.

Common dolphins are generalist feeders preying on locally abundant shoaling epipelagic fishes and cephalopods [29, 30]. Fisheries-independent information on the abundance of small pelagic species in the Azores is scarce. Therefore, to investigate the effect of prey availability on the probability of dolphins interacting with the tuna fishery, we used landings statistics of locally abundant species that likely serve as dolphin prey. Landing data of *Boops boops*, *Sardina pilchardus*, *Scomber colias* and *Trachurus picturatus* (available through Lotaçor) were pooled together for each month of the study period and used as a proxy for prey abundance in the region.

### Analysis of data

To analyze spatial trends in common dolphin interaction we divided the number of fishing events with interaction by the total number of fishing events for each cell of a 10x10 nm grid overlaying the study area. The spatial distribution of interaction rates is presented per 1000 fishing events.

The temporal pattern in common dolphin interaction with the tuna fishery was investigated by fitting a Generalized Linear Model (GLM) with a binomial error distribution and identity link function for the response variable. The response variable was the presence or absence of dolphin interaction during a given fishing event. To investigate if there was a decreasing trend in the probability of interaction over time, a model with estimated annual means across all years was compared to a model where year was included as a continuous predictor [31].

Generalized Additive Models (GAMs) were used to examine the effect of variables related to the fishing effort, fishing operations, tuna catches and environment on the occurrence of common dolphin interaction on a given fishing event (Table 1). The variables tuna weight (kg) and average weight of individual tuna (kg) were found to be autocorrelated. Models including any one of these variables provided a worse fit than models using average tuna size and number of tuna. The variables fishing duration, distance to coast, number of tuna caught, and average length of individual tuna were log transformed (+1 for covariates including zero values). The GAM was used to account for non-linear effects of predictors. The GAM predicting the presence/ absence of interaction also used a binomial error distribution with an identity link function.

**Table 1:** Summary of explanatory variables for the GAM predicting presence/absence of dolphin interaction and the NB GAM predicting effect of interaction on tuna catches.

Response variable	Category	Explanatory variables	Type
Presence/absence of common dolphin interaction	Environmental variables	Sea surface temperature (°)	Continuous
		Depth (m)	Continuous
		Distance shore (km)	Continuous
		Prey abundance (t)	Continuous
	Fishing operations	Hour of day	Continuous
		Gear	Categorical
		Latitude, Longitude	Continuous
		Type of baitfish	Categorical
	Fishing effort	Fishing duration (h)	Continuous
		Number of poles	Continuous
	Catch	Number of tuna	Continuous
		Tuna species	Categorical
		Average tuna size (cm)	Continuous
Number of Tuna captured	Environmental variables	Sea surface temperature (°)	Continuous
		Depth (m)	Continuous
		Prey abundance (t)	Continuous
	Fishing operations	Hour of day (h)	Continuous
		Type of baitfish	Categorical
	Fishing effort	Fishing duration (h)	Continuous
		Number of poles	Continuous
	Dolphin interaction	Yes /No	Categorical

A Negative Binomial GAM with a log link function was used to estimate the effect of common dolphin interaction on tuna catches. The response variable consisted of counts of skipjack, yellowfin, albacore and bigeye tuna captured per fishing event. The explanatory variables considered included only covariates expected to be influential on tuna catches: SST, depth, prey abundance, hour of day, fishing duration and number of poles were fitted as continuous variables; while type of baitfish used and presence of common dolphin interaction were categorical variables (Table 1). To adjust for differences in fishing effort an “offset” variable (log of fishing duration) was applied.

Before fitting the models, homogeneity, potential outliers and amount of zeros were analysed with Cleveland dotplots, boxplots, and frequency plots. Variance Inflation Factor analysis and pairwise correlations were computed between all variables to check for collinearity. Variables with the highest Akaike Information Criterion (AIC) score were excluded. A backward stepwise selection procedure was used to identify the best fitting model using the AIC value. Model validation was applied on the best fitting model to verify the underlying assumptions [32].

To calculate the catch loss caused by the interaction of common dolphins, we estimated the number of fish that would have been caught in the absence of interaction, using the best fitting negative binomial GAM. Catches per fishing event were predicted by year and area and the common dolphin interaction variable was set to “0” [31]. Differences between the observed and predicted number of tuna fish captured were used to compute the percentage catch variation per year for each fishing area.

The predicted number of fish lost due to dolphin interaction was then multiplied by the average weigh of individual fish landed on a given year and fishing area [18] to calculate the catch reduction in weight and obtain a rough estimate of the economic loss.

All analyses were conducted using R Statistical Computing Software (version 3.2.1).

## Results

### Frequency of dolphin interaction

A total of 2387 fishing trips were monitored during which 23175 fishing events were recorded. Cetaceans were present in about 6% (n = 1487) of the fishing events. Sixteen cetacean species were recorded during tuna fishing activities. Common dolphin was the most frequently observed species (62%), followed by Atlantic spotted dolphins (*Stenella frontalis*) (18%) and bottlenose dolphins (*Tursiops truncatus*) (9%).

Dolphin interaction with the fishery occurred in 2.8% (n = 673) of the fishing events. Common dolphins were responsible for 66% of the reports of interaction, followed by spotted dolphins (19%) and bottlenose dolphins (10%).

**Fig 1:**
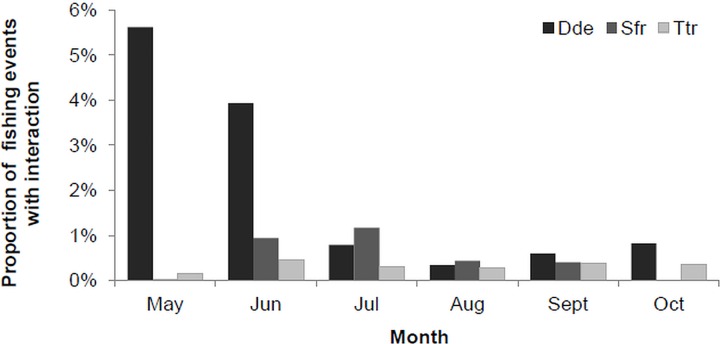
Proportion of fishing events with interaction with common dolphins (Dde), spotted dolphins (Sfr) and bottlenose dolphins (Ttr) per month from 1998 to 2012.

Interaction rates varied by month and species, with a higher proportion of fishing events with interaction in May and June, the majority of which involved common dolphins. From July to October the proportion of events with interaction dropped to <1% for the three species (Fig 1).

### Spatial and temporal patterns in common dolphin interaction

**Fig 2:**
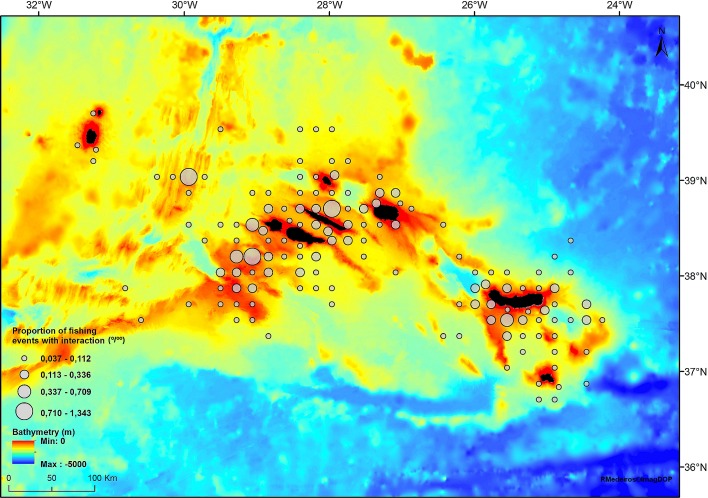
Spatial distribution of fishing events with common dolphin interaction (per 1000 fishing events). The size of the grey circles is proportional to the rate of fishing events with dolphin interactions. Reprinted from *Imag*DOP under a CC BY license, with permission from Ricardo Medeiros, original copyright 2015.

Common dolphin interaction was reported in all three groups of islands. Interaction rates were higher in the central and eastern groups with only a few cases reported in the western group where little fishing effort occurred (Fig 2).

**Fig 3:**
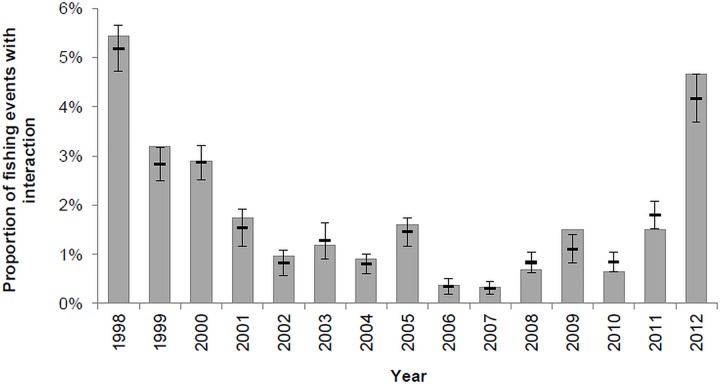
Observed (vertical grey bars) and estimated (horizontal black bars) proportion of fishing events with common dolphin interaction per year. The standard deviation of the estimates is represented by the vertical whiskers.

There were significant differences among years in the probability of common dolphins interacting with the tuna fishery (p<0.001) but there was no evidence of a decreasing trend over time, as indicated by the higher AIC score of the model testing for a linear effect between year and interaction probability (in the scale of the link function). In fact, probability of dolphin interaction in the tuna fishery decreased from 5.2% to 0.3% between 1998 and 2006, but increased again in the latter period of the study reaching a secondary maxima in 2012 (4.2%) (Fig 3).

### Factors affecting probability of common dolphin interaction

Modeling results suggested that fishing duration, SST and prey abundance were the most important predictors of the probability of dolphin interaction with the tuna fishery. Hour of day, number and average size of individual tuna, and fishing location were also significant predictors of dolphin interaction but showed a weaker effect. Depth, distance to shore, gear, number of poles, tuna species and type of baitfish used had no significant effect on interaction probability (p>0.05 for all) (Table 2).

**Table 2:** Summary of parameter estimates from the best-fitting GAM predicting probability of common dolphin interaction in the fishery.

Response variable	Explanatory variable	Parametric coefficients				Non-parametric smoothers		
		ß	SE	Z	*P*	edf	Χ^2^	P
Interaction probability	Intercept	-6.439	0.767	-8.395	<0.001			
	Number of tuna	-0.108	0.041	-2.637	0.008			
	Average tuna size	0.420	0.170	2.474	0.013			
	s(Hour of day)					5.948	29.92	<0.001
	s(Fishing duration)					1.852	153.07	<0.001
	s(long,lat)					15.170	45.74	<0.001
	s(SST)					5.315	106.82	<0.001
	s(Prey abundance)					8.658	77.74	<0.001

ß = Parameter estimate, SE = Standard error, edf = effective degrees of freedom.

The final GAM model explained 21.8% of the deviance and included number and average size of individual tuna as parametric terms, and smoothers for the remaining variables. Probability of interaction was higher during early morning and late afternoon. Note that the standard errors of the estimates were wider from 20:00–6:00 hrs because there was little fishing activity at night. Likelihood of interaction increased almost linearly with duration of fishing events. As already seen from the spatial distribution of fishing events with interaction, the probability of dolphins interacting with the fishery increased towards the central group of islands (between 36° and 38° longitude).

**Fig 4:**
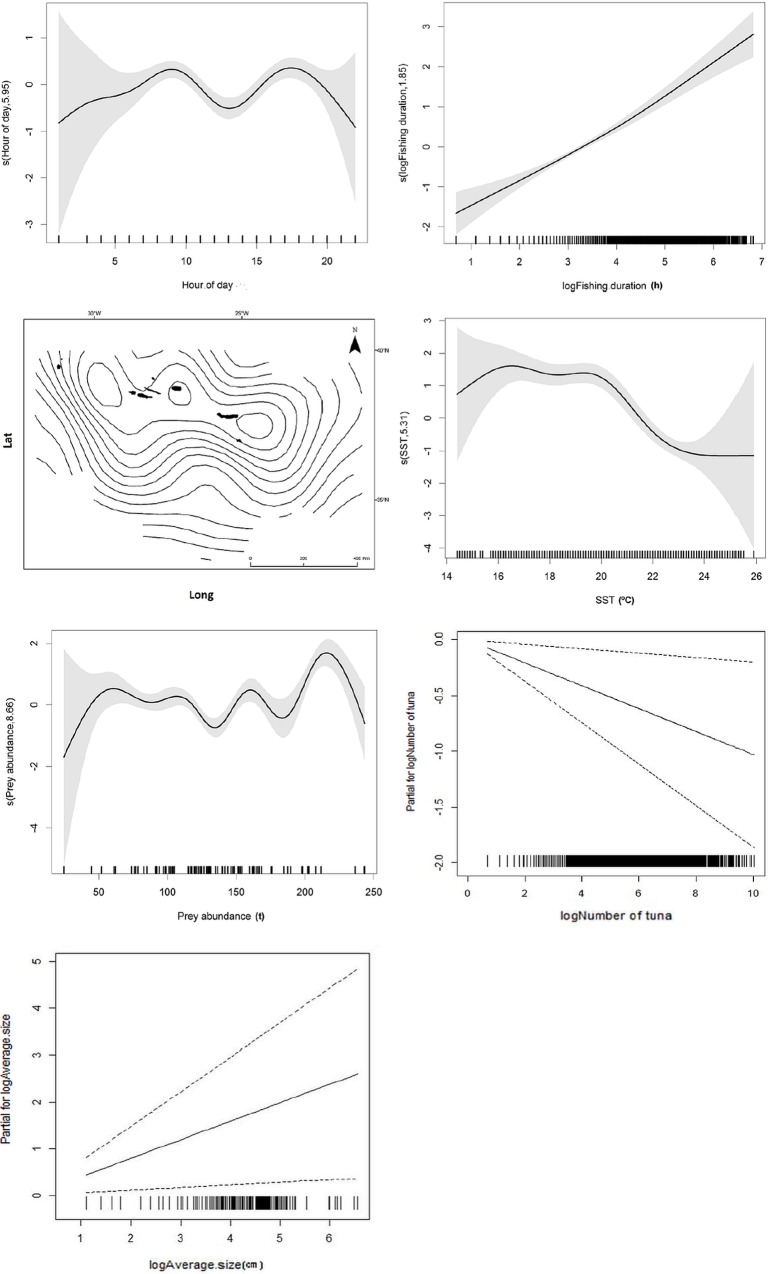
Smoother estimates (solid line) for the predictors hour of day, fishing duration, spatial location (latitude/longitude), SST, prey abundance and parametric terms for the number and average size of individual tuna obtained by the GAM predicting probability of common dolphin interaction. The grey shading indicates approximate 95% confidence bands. Tick marks on the x-axis are sampled data points.

Tuna fishing occurred at times of the year when SST ranged from 14.4°C to 26°C and interaction was higher for temperatures between 17°C and 20°C. The probability of dolphin interaction in relation to prey abundance was also non-linear, with increasing probabilities for values higher than 200 tons. Common dolphin interaction increased linearly with average size of tunas and decreased with the number of tunas caught (Fig 4).

### Factors affecting probability of tuna catch and catch reduction

Negative binomial GAMs were developed to investigate changes in catches for each tuna species associated with common dolphin interaction. Final models for each tuna species explained between 42.3% and 60.9% of the deviance (theta values <1.2) (Table 3).

**Table 3:** Summary of parameter estimates from the best-fitting negative binomial GAM for the number of tuna caught for each tuna species.

	Explanatory variable	Parametric coefficients					Non-parametric smoothers		
		ß		SE	z	*P*	edf	Χ^2^	P
*K*. *pelamis*	s(Hour of day)						6.871	86.13	<0.001
	s(Depth)						7.363	163.08	<0.001
	s(SST)						4.859	105.39	<0.001
	s(N° poles)						7.554	5332.58	<0.001
	s(Prey abundance)						8.712	336.82	<0.001
*T*.*alalunga*	Type of baitfish (mixed)	-0.183		0.076	-2.40	0.02			
	Type of baitfish (*S*. *pilchardus*)	-0.489		0.105	-4.67	<0.001			
	S(SST)						5.610	37.65	<0.001
	S(N° poles)						4.461	116.30	<0.001
	s(Prey abundance)						7.556	57.86	<0.001
*T*. *albacares*	s(Depth)						2.040	10.34	0.011
	s(SST)						3.282	19.14	<0.001
	s(N° poles)						1.148	49.21	<0.001
*T*.*obsesus*	Prey abundance	0.001		0.0003	4.38	<0.001			
	Dolphin interaction	-0.358		0.059	-6.05	<0.001			
	s(Hour of day)						5.803	18.04	0.011
	s(Depth)						7.558	219.77	<0.001
	s(SST)						5.848	278.46	<0.001
	s(N° poles)						7.621	1029.34	<0.001

ß = Parameter estimate, SE = Standard error, edf = effective degrees of freedom

**Fig 5:**
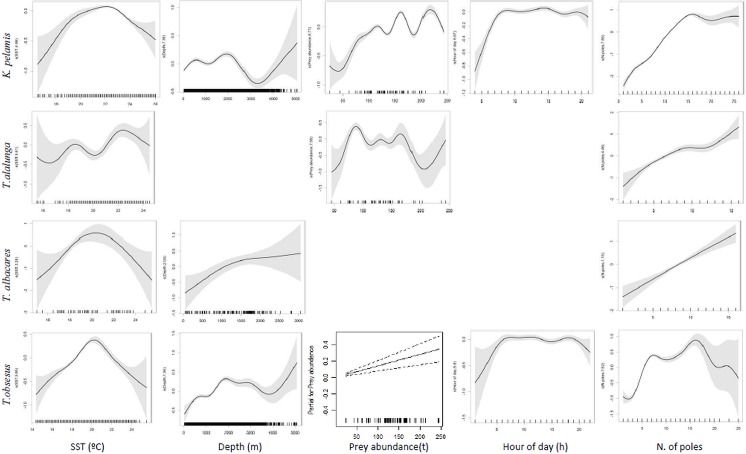
Smoother estimates (solid line) for the predictors SST, depth, prey abundance, hour of day and number of poles obtained by negative binomial GAM (NB GAM) for predicting changes in tuna catches with common dolphin interaction. The approximate 95% confidence envelopes are indicated (grey shading). Tick marks on the x-axis are sampled data points.

SST and number of fishing poles had a significant effect in the catches of all tuna species. Captures of yellowfin and bigeye tuna increased when temperatures reached 20°C, while captures of skipjack and albacore tuna were higher at temperatures ranging between 20°C and 22°C. Tuna captures also increased with the number of fishing poles used. Depth affected significantly the captures of skipjack, bigeye and yellowfin tuna, with increased captures for depths of about 2000 m. Skipjack, albacore and bigeye tuna catches were higher with increased prey abundance. The type of baitfish used was only significant in the captures of albacore, which showed a preference for blue jack mackerel. Catches of skipjack and bigeye tuna were higher during the day than at night, but hour of day had no influence on takes of other tuna species (Fig 5).

Common dolphin interaction had no significant effect on the catches of albacore, skipjack and yellowfin tuna. In contrast, the best fitting model for bigeye tuna predicted a decline of about 36% in the number of tunas captured in the presence of dolphin interaction for the whole study period (1998–2012) (Table 3). Based on this model, we estimated the number of tunas that would have been captured in the absence of interaction during fishing events for bigeye (n = 7606).

**Fig 6:**
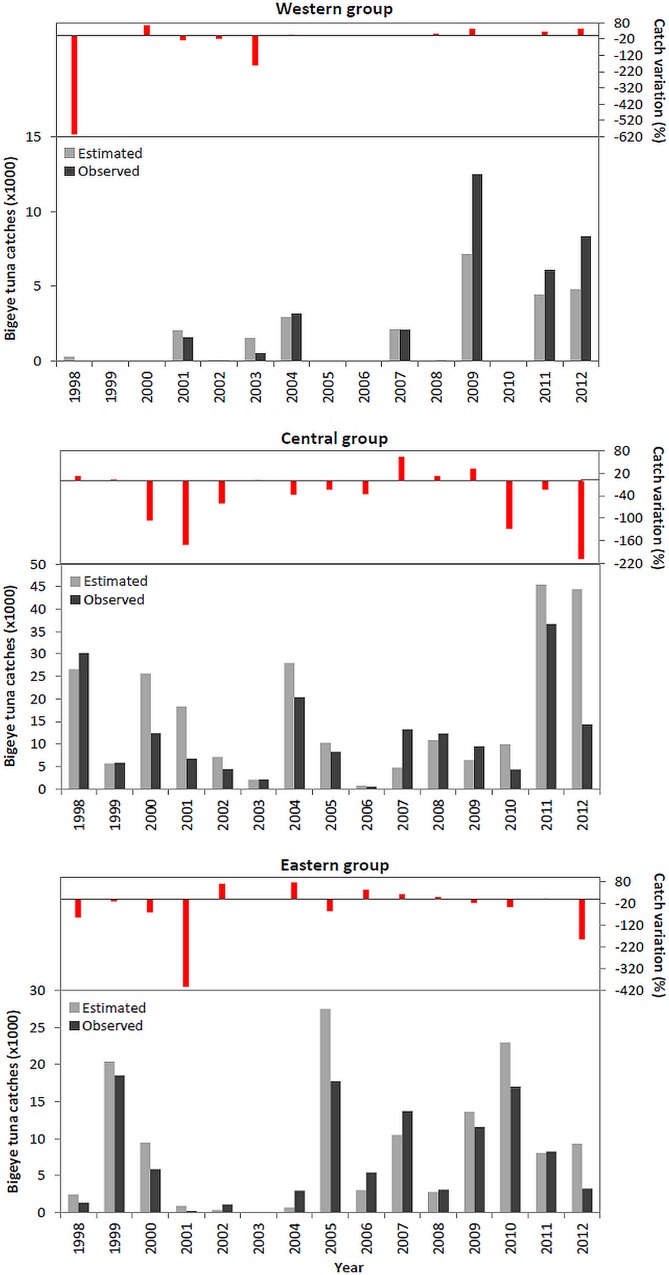
Observed (dark grey) and model-estimated (light grey) average annual catches (in number) of bigeye tuna in the absence of common dolphin interaction and percentage of catch variation between the observed and model-estimated catches (in number) for each group of islands.

Impact of dolphin interaction in the capture of bigeye tuna varied both by year and area with no consistent annual pattern across groups of islands (Fig 6). The highest catch losses were estimated in 1998 in the western islands and in 2001 in the eastern islands, with a 607% and 403% reduction in the number of bigeyes captured, respectively. However, in almost half of the years, predicted catches in the absence of dolphin interaction were lower than actual catches. Model-based estimates suggest that the negative effect on bigeye catches only occurred in the central and eastern islands, with median annual losses in number of fish captured of 24% and 14%, respectively. In contrast, in the western group, observed catches with dolphin interaction had 7% more tuna than model-estimated catches in the absence of interaction.

Based on the average price of bigeye tuna per year in the Azores, the value of fish lost from interaction with common dolphins is estimated at €304 thousand per year.

## Discussion

Dolphin interaction in the pole-and-line tuna fishery was generally low occurring in about 3% of all fishing events. Common dolphins were responsible for most of the interactions recorded, in particular during the months of May and June. While rates of interaction by common dolphins declined in July, spotted dolphin interaction increased in June reaching a peak in July. Common dolphins are sighted in the Azores year-round and are one of the most abundant cetacean species in the area [33, 34]. However, Silva *et al*. [34] described a clear seasonal pattern in the occurrence of the species, with encounter rates decreasing in the summer and autumn, coincidental with the increase in encounter rates of spotted dolphins. The reasons behind the inverse trend in seasonal occurrence of the two species in the area remain unknown. However, it likely explains the monthly variations in interaction rates for each species, as well as the overall decreasing trend in interaction rates over months, as common dolphins became less abundant in the area. In addition, the tuna species seasonally present in the area may also influence the variation of the monthly interaction rate, as discussed below.

Using a longer time series allowed us to verify that common dolphin interaction did not maintain the decreasing trend reported by Silva *et al*. [23]. Interaction rates decreased from 1998 to the mid-2000s increasing again after this and reaching values similar to those recorded in the beginning of the monitoring programme. Despite this general tendency, the frequency of common dolphin interaction varied significantly from year to year. The reasons for this inter-annual variation can be explained by the modelling results. The final model explaining the probability of common dolphin interaction contained the following factors as significant variables, in decreasing order of importance: fishing duration, SST, prey abundance, hour of day, fishing location, number and average size of individual tuna.

The duration of fishing events was an important factor as common dolphin interaction increased with longer fishing events. Longer fishing events should incite common dolphins to remain in the fishing area to feed on the live bait that fishermen periodically throw to sea, thus increasing the chances of interaction. Concentration of prey may also attract increasing numbers of dolphins to the fishing area. In prawn trawl fisheries, for example, dolphins were regularly observed following trawler vessels to forage on discarded fish [35–37]. Also, dolphin presence increased near fish farms since they aggregate more prey, making it more energetically efficient for animals to spend time in that area [38, 39].

Common dolphins were more likely to interact with the pole-and-line fishery during early morning and afternoon, corresponding to the periods of intense fishing effort. Dolphins interacted more frequently with the fishing at lower sea surface temperatures, reflecting the increased relative abundance of the species in early spring and the decline from late spring onwards [34], when seawater temperatures get warmer.

Location also influenced common dolphin interaction with increased rates in the eastern and central groups of islands compared to the western islands. We believe that this pattern may be explained by the interplay between the spatial distribution of fishing effort and the temporal occurrence of common dolphins in the region. During spring and early summer, when common dolphins are known to be more abundant in the Azores [34], fishing effort was mainly concentrated in the central and eastern groups of islands, explaining the higher interaction rates in these areas. Fishing in the western islands only started in mid-summer, after the sudden decline in common dolphin relative abundance. Within the central and eastern groups of islands, fishing events that took place in the vicinity of seamounts were the most affected by dolphin interaction. Common dolphins are frequently sighted around seamounts in the Azores and possibly use these remote sites as feeding areas, taking advantage of the localized abundance of prey items [19, 40, 41].

The model also indicates that prey abundance, represented by the combined landings of four small pelagic fishes increased the likelihood of dolphin interaction. This may seem counterintuitive as we would expect dolphins to interact less frequently with the tuna fishery at times of greater prey availability in the area. We believe these results may be explained by the temporary association between common dolphins and bigeye tuna. Association between the two species accounts for over 73% of cetacean-tuna associations recorded in the Azores [42]. As mentioned above, the two species overlap in their seasonal abundance in the area, possibly because they have similar prey preferences and explore the same feeding areas [43, 44]. Higher prey abundances would presumably attract common dolphins and tunas to forage in the same areas. Prey is driven to the surface by tuna and further concentrated by common dolphins, resulting in the formation of a tight ball of forage fish close to the surface allowing dolphins and other species like seabirds and sharks to feed at the same time [10, 19, 22, 45]. The feeding frenzy could then attract fishing vessels to the area where dolphins co-occur with tuna schools, thus increasing the opportunities for dolphins to interact with the fishery.

Despite the strong relationship between prey abundance index and interaction probability, these results should be interpreted with caution because landing data may be a poor proxy for prey abundance in the region. First, a portion of the catch of small pelagic fishes may not be landed, as species of low economic value (e.g., European pilchard and bogue) are sometimes discarded or used as bait. Second, baitfish species captured by the tuna fishery are not included in the landing statistics. Still, in the absence of data from fisheries research surveys, landings could provide a rough indication of fluctuations in prey availability. Another potential caveat is that the index used here only includes data from a small number of potential prey species available for common dolphins. Although these species are very abundant locally [20, 46], common dolphins are known to consume a variety of prey items and are well adapted to cope with changes in prey species availability [47, 48]. In the future, prey biomass data from biogeochemical models [49] should be incorporated in the investigation of dolphin-fisheries interactions.

The association between common dolphins and the bigeye tuna could also explain why dolphins interacted more frequently in fishing events with larger tunas. Fishing events with bluefin tuna were uncommon in our dataset, and bigeyes are the second largest tuna species captured in the Azores. It may be easier for larger fish to form mixed groups with dolphins as their swimming speed may be comparable [50]. Probability of common dolphin interaction decreased with increasing number of tuna captured. The most plausible explanation is that dolphin interaction leads to the premature interruption of fishing activity resulting in fishing events with fewer tunas.

Common dolphin interaction resulted in an overall decline in bigeye tuna catches, while for the other tuna species no effect was detected. This result may simply be explained by a higher frequency of interactions with bigeye than with other species. Common dolphins were frequently observed feeding on live bait. Competition for the live bait between dolphins and tuna may drive tuna away from the fishing area. Cetaceans are known to interact with the tuna fishery, mainly by depredating tuna caught in the fishing gear. Off southern and south-eastern Brazil, killer whales (*Orcinus orca*) remove tuna (*Thunnus* spp.) caught in pelagic longlines [51]. In Hawaii false killer whales (*Pseudorca crassidens*) have been observed depredating the catch, as well as the bait used in deep-set pelagic longline fisheries [52].

The impact of dolphin interaction on bigeye catches was, nonetheless, different in each fishing area. In the central and eastern islands, dolphin interaction resulted in median annual catch losses of 24% and 14%, respectively, whereas in the western islands the model predicted lower catches in the absence of interaction. It is possible that the model developed for the whole Azorean EEZ failed to capture fine-scale differences in fishing practices or tuna-environment relationships, thereby underestimating bigeye catches in the western islands. As sample size increases with the continuation of POPA programme, it may be possible to develop separate models for each area.

Also, in addition to dolphin interaction, environmental factors and factors related to fishing operations strongly influenced bigeye catches, contributing to substantial differences in the estimated impact of dolphin interaction between years. Some of these factors may contribute to catch changes comparable to those of dolphin interaction. For instance, the model predicts that a 2°C decrease in SST could lead to a reduction in captures of bigeye of about 18%.

The interaction of common dolphins with the bigeye tuna fishery resulted in an estimated economic loss of €304 thousand per year. Considering that the pole-and-line tuna fishery was worth approximately €7 million per year during this period, this represents approximately 4% of the value of tuna landed. Cruz *et al*. [53] estimated that Risso´s dolphin depredation in the artisanal squid fishery in São Miguel island, Azores (where most of the fishery takes place) affected 33% of the fishing events from 2009–2011, representing an estimated loss of €50 thousand per year, 1.9% of total landed value of this fishery over this time. Thus, compared to Risso´s dolphin interaction in the squid fishery, common dolphin interaction in the tuna fishery is less frequent but has a greater economic impact. Although, these figures are well below estimates of economic loss from cetacean interaction in fisheries elsewhere like in the Balearic Islands and the Crozet islands [1, 54].

Common dolphin interaction in the tuna fishery may be driven by a number of ecological factors not considered in this study. Variability in common dolphin distribution and abundance in the fishing region is expected to be one of the most important factors affecting interaction rates but this information is not available for the study site. An attempt was made to use common dolphin encounter rate derived from POPA data as a proxy for the species abundance but this variable was not retained in any of the models, probably because it was not independent from dolphin presence and interaction in the fishing activity. Information on prey density may also provide further insights into common dolphin and tuna distribution within this region. Although tuna species was not a significant predictor of the probability of interaction, common dolphins frequently associated with bigeye tuna and catches of this tuna species were affected by dolphin interaction. Therefore information on bigeye movements, in addition to dolphin movements, should also improve our understanding of interaction patterns.

## Conclusions

Identification of drivers and consequences of cetacean-fisheries interactions is fundamental to assessing the need to establish measures to reduce impacts on cetacean populations and on commercial fisheries, as well as to evaluate the feasibility of such measures. Except for the research previously conducted in the Azores, that produced a preliminary characterization, we are not aware of studies investigating cetacean interaction in the pole-and-line tuna fishery. This study fills in this gap, by providing important information on fishing practices and ecological processes favouring the interaction of small dolphins in this fishery.

Common dolphins are opportunistic feeders that take advantage of locally abundant prey. In the Azores they often form temporary foraging associations with tunas especially bigeye, possibly to increase feeding success. Thus, common dolphins and tunas exploit similar habitats and frequently co-occur in the same areas. In addition, the pole-and-line tuna fishery in the Azores relies on the use of live bait. Live bait represents an easy and readily available food source for common dolphins and it is not surprising that dolphins are attracted to fishing vessels to prey on it. Not surprisingly, interaction was not associated with a specific gear, fishing area or time of the year but was mainly correlated with variables representing fishing effort, distribution of tuna and possibly dolphins. In these circumstances, there are no easy solutions to reduce interaction rates.

Several studies have investigated mitigation measures to reduce cetacean interaction including changes in fishing practices, gear modifications, acoustic deterrents and harassment devices, and mechanisms to prevent access to the catch [55–58]. Given the specificities of the pole-and-line tuna fishing in the Azores, and the ecology of common dolphins and tuna, the only measure that could potentially reduce interaction would be to keep dolphins away from the fishing area using acoustic devices. However, the effectiveness of acoustic deterrents at decreasing cetacean interaction is still unclear [57, 59, 60]. Recently, Cruz *et al*. [53] showed that depredation rates by Risso’s dolphins on the squid fishery in the Azores did not decrease with the use of various pingers. Moreover, the effect of these devices on tuna behaviour would need to be investigated before this mitigation measure could be considered.

Still, common dolphin interaction rates with the pole-and-line tuna fishery are relatively low and catch reductions are moderate and only evident for bigeye tuna. Furthermore, there is no evidence that dolphin interaction is growing or becoming more severe over time. Considering the well-established negative effects of anthropogenic noise, the use of any type of acoustic device should be carefully weighted and be preceded of studies to assess their impact and effectiveness. It will also be fundamental to determine the costs of potential mitigation measures, as they may end up endangering the economic viability of such a small-scale fishery. In the meantime it would be crucial to inform fishermen about real impacts of dolphin interaction and implement educational campaigns about sustainable use of marine ecosystems, as these may prove more effective to manage cetacean-fisheries conflicts in the long term.
